# Cognitive Bias Modification Training Targeting Fatigue in Patients With Kidney Disease: Usability Study

**DOI:** 10.2196/43636

**Published:** 2023-05-29

**Authors:** Jody Geerts, Marcel Pieterse, Goos Laverman, Femke Waanders, Nicole Oosterom, Jacqueline Slegten, Elske Salemink, Christina Bode

**Affiliations:** 1 Centre for eHealth & Well-being Research Section Psychology, Health and Technology University of Twente Enschede Netherlands; 2 Department of Internal Medicine Division of Nephrology Ziekenhuisgroep Twente Hospital Almelo Netherlands; 3 Biomedical Signals and Systems research group Faculty of Electrical Engineering, Mathematics and Computer Science Twente University Enschede Netherlands; 4 Department of Internal Medicine Isala Hospital Zwolle Netherlands; 5 Department of Clinical Psychology Utrecht University Utrecht Netherlands

**Keywords:** cognitive bias, patient perspective, qualitative study, nephrology, fatigue, vitality, acceptability, applicability, usability, design

## Abstract

**Background:**

Fatigue is an important symptom for many patients, including patients with kidney disease. Cognitive biases, such as attentional bias and self-identity bias, are thought to influence fatigue. Cognitive bias modification (CBM) training is a promising technique to counter fatigue.

**Objective:**

We aimed to evaluate a CBM training among patients with kidney disease and health care professionals (HCPs) and assess acceptability and applicability in the clinical setting using an iterative design process to evaluate expectations and experiences with the training.

**Methods:**

This was a longitudinal, qualitative, and multiple stakeholder–perspective usability study in which we interviewed end users and HCPs during the prototyping phase and after the end of training. We conducted semistructured interviews with 29 patients and 16 HCPs. The interviews were transcribed and analyzed thematically. Next to a general evaluation of the training, the acceptability of the training was evaluated using the Theoretical Framework of Acceptability, and applicability was assessed by evaluating obstacles and solutions for implementation in the kidney care setting.

**Results:**

Generally, participants were positive about the training and its applicability. The biggest negatives were doubts about effectiveness and annoyance about the repetitive character of CBM. Acceptability was judged with a mixed evaluation, with a negative evaluation of perceived effectiveness; mixed results for burden, intervention coherence, and self-efficacy; and positive results for affective attitude, ethicality, and opportunity costs. Barriers for applicability were patients’ varying computer skills, subjectivity of fatigue, and integration with regular treatment (eg, the role of HCPs). Possible solutions included assigning representatives among nurses, offering training on an app, and providing assistance via a help desk. The iterative design process, including repeated waves of testing user expectations and experiences, yielded complementary data.

**Conclusions:**

To the best of our knowledge, this study is the first to introduce a CBM training targeting fatigue. Furthermore, this study provides one of the first user evaluations of a CBM training, both among patients with kidney disease and their care providers. Overall, the training was evaluated positively, although acceptability showed mixed results. Applicability was positive although barriers were identified. The proposed solutions require further testing, preferably following the same frameworks, as the iteration in this study contributed positively to the quality of the training. Therefore, future research should follow the same frameworks and consider stakeholders and end users in eHealth intervention design.

## Introduction

### Fatigue Bias

Fatigue has been recognized as one of the most frequent and important symptoms of many illnesses and has been rated as one of the key factors leading to a decrease in quality of life [1]. Patients with chronic kidney disease (CKD) are no exception: in particular, in patients dependent on chronic hemodialysis, the prevalence of severe fatigue is very high (53.3% [2]). Psychological processes are important determinants of fatigue. Even in patients undergoing hemodialysis, where physical factors are considered as strong determinants of fatigue severity, psychological aspects, such as stress, negative beliefs about fatigue, and unhelpful behaviors, predict 36.4% of fatigue severity [3]. Furthermore, it has been shown that biases in more automatic processing of information (implicit cognitive biases) are important in predicting and maintaining factors for multiple illness-related symptoms, including fatigue. For instance, Hughes et al [4] showed that patients with chronic fatigue syndrome consistently show an attentional bias toward health-threatening cues compared with healthy controls. Other biases also play a role; for example, identity bias (a distorted perception of the self) and memory bias (distortions in memory retrieval processes) were found to be related to pain severity [5-7].

### Cognitive Bias Modification

Cognitive bias modification (CBM) is a novel technique that targets cognitive biases by directly retraining them using simple computer tasks [8]. Although not confirmed by all studies in this field [9,10], CBM has produced promising results in countering pain [11,12]; depression; trait and social anxiety [13-17]; alcohol dependency and addiction [11,15,18-22]; fear of cancer recurrence [23]; eating disorders [11,24]; and unhealthy consumer behavior such as cigarette smoking, alcohol use, and unhealthy eating [25]. Because CBM is based on simple computer tasks, it is an easy, accessible, and inexpensive option compared with other interventions. Furthermore, CBM is thought to be especially useful in stressful situations because it is directed at more unconscious or implicit processes and requires less active reflection from the participant [13,26]. These factors make CBM a promising intervention for treating fatigue symptoms.

The CBM training in this study targets 2 different biases, namely attentional bias (having more attention for symptoms, ie, hypervigilance) and self-identity bias (ie, using symptoms to identify oneself, eg, “I am a tired person”). To correct self-identity bias, an implicit association task (IAT) paradigm can be used [27] and changed into a training paradigm [28]. Patients are trained to pair positive stimuli (good and happy) with *self* (I and me) and negative stimuli (bad and disaster) with *other* (they and them). Through a series of quick response tasks, novel associative links are established within the memory and gradually automatized. Similarly, in attentional CBM, participants are guided to—against their habit—ignore threatening cues and instead direct their attention to positive cues. On the basis of the visual probe task (VPT) paradigm [29], participants see 2 words appearing simultaneously on the computer screen: a positive or neutral word and a threatening word. After brief exposure to the pair of words, participants have to respond to the target that systematically appears in the location of the positive stimulus, training participants to ignore the threatening words and direct their attention to positive cues [30].

### User’s Perspective

Owing to these simple computer tasks, CBM is expected to be an attractive intervention for multiple patient populations. However, very little is known about the actual CBM experience of patients. To the best of our knowledge, only 1 qualitative study exists: Beard et al [31] revealed mixed reactions from primary care patients with social anxiety using CBM. Although most participants were positive about the rationale behind CBM and identified with the negative bias described, some were skeptical and not convinced of the relevance, purpose, and benefits of the specific tasks. Moreover, some disliked the repetitive and boring nature of CBM [31]. Thus, even though participants seem willing to accept the idea of CBM, the simplicity that makes CBM a promising intervention appears at the same time as a barrier for participants to engage in and complete CBM treatment.

Furthermore, although good examples can be found [32,33], the implementation of eHealth interventions can be problematic, despite promising results in clinical trials, often owing to the lack of digital skills or knowledge of the eHealth app in patients and health care professionals (HCPs) [34,35]. Therefore, in eHealth development, it is important to take users’ perspectives into account as it improves usability, prevents the design from having unnecessary features, prevents resistance and dropout, and can subsequently prevent spending money on poorly fitting designs [36,37]. Thus, although neglected by most studies on CBM, patients’ and HCPs’ perspectives are important factors to consider in the development of CBM interventions as they can improve acceptability, usability, and successful implementation [38,39].

### Acceptability and Sustainability

Acceptability and successful implementation have been receiving increasing attention in health psychology lately [40]. Until recently, no definition of acceptability was included [41]. Therefore, Sekhon et al [42] used their review to develop the Theoretical Framework of Acceptability (TFA) and defined acceptability as “a multi-faceted construct that reflects the extent to which people delivering or receiving a healthcare intervention consider it to be appropriate, based on anticipated or experiential cognitive and emotional responses to the intervention.” Moreover, they identified seven components: (1) affective attitude; how participants feel about the intervention, (2) burden; the amount of effort perceived to be required for intervention participation, (3) ethicality; whether the intervention has a good fit with the participant’s values, (4) intervention coherence; whether the participant understands the intervention and its mechanisms, (5) opportunity costs; whether profits, benefits, or values have to be sacrificed to engage in the intervention, (6) perceived effectiveness; whether the intervention is perceived to be likely to achieve its purpose, and (7) self-efficacy, the participant’s confidence about performing the behavior required for intervention participation [41,42].

Moreover, multiple frameworks recognize user perspective and acceptability as important factors in their aim for the long-term implementation of interventions and focus on sustainability. The Dynamic Sustainability Framework [43], for instance, emphasizes sustainability with three focus points: (1) ongoing learning and problem-solving, (2) a focus on the fit between interventions and the multilevel contexts they are to be implemented in and a continuous adaptation to that fit, and (3) a change in expectations of researchers from accepting diminishing outcomes over time to expecting ongoing improvements. Building on this, the Center for eHealth Research and Disease Management (CeHRes) Roadmap [44] provides a framework for an iterative development process for eHealth apps, considering the human, contextual, and technological factors that increase the chance of an intervention being a good fit and reaching its goals [45]. By emphasizing a dynamic and iterative development process that considers multiple perspectives and contexts, these frameworks aim to achieve more sustainable and successful interventions. In line with these frameworks, this study used a longitudinal iterative process to develop a CBM training targeting fatigue. This training was evaluated by multiple stakeholders, namely patients with CKD and their HCPs.

### Aims

The primary aim of this study was to evaluate the CBM training targeting fatigue with patients suffering from CKD and nephrology professionals. The interviews were conducted at 2 developmental stages: the prototype stage (expectations) and after an 8- to 9-week study with CBM training (experiences). With a combination of multiple stakeholder perspectives and developmental stages, we aimed to provide a comprehensive evaluation of the CBM training. Specifically, acceptability and applicability in the clinical setting were evaluated, and obstacles and possible solutions were discussed.

## Methods

### Participants

The prototype phase included Dutch-speaking adult patients with CKD, nephrologists, nurse practitioners, dialysis nurses, and social workers. At the evaluation phase, Dutch-speaking adult patients with CKD who had reported moderate to severe fatigue and had adequate visual capabilities to operate a computer and basic internet skills were included. Patients scheduled to undergo kidney transplantation within 3 months or patients with any somatic or psychiatric comorbidity that may impede patient adherence to the study protocol were excluded. All invited patients participated in the prototype phase. During the evaluation phase, 6 (25%) out of the 24 invited patients did not agree to participate in the interviews because of hearing problems (n=2), lack of energy (n=1), working full time (n=1), having a hectic time during the COVID-19 pandemic (n=1), or the preference to ask others first because of other commitments (n=1).

In the prototype phase, 21 interviews were conducted with 10 professionals (4 nephrologists, 2 nurse practitioners, 2 dialysis nurses, and 2 social workers) and 11 patients (5 patients in predialysis stage [CKD 4 and 5] and 6 patients dependent on dialysis [CKD 5D]). Half of the patients (6/11, 55%) reported having fatigue symptoms. The patients’ ages ranged from 27 to 80 (mean 65, SD 14.8) years, and 8 patients were female. During the evaluation phase, 24 interviews were conducted with the 6 involved professionals (2 nephrologists, 3 nurse practitioners, and 1 dialysis nurse) and 18 patients (8 patients with CKD stages 4 and 5 and 10 patients with CKD stage 5D, of which 3 underwent peritoneal dialysis). During the evaluation phase, the patients’ ages ranged from 45 to 83 (mean 64, SD 9.9) years, and 8 patients were female. In Table 1, the number and characteristics of participants are depicted for the 2 phases.

**Table 1:** The number and characteristics of participated patients and health care professionals (HCPs) at the prototype phase and evaluation phase.

Characteristic		Patient		HCP	
		Prototype (n=11)	Evaluation (n=18)	Prototype (n=10)	Evaluation (n=6)
**CKD^a,b^, n (%)**					
	Stages 4-5	5 (45)	8 (44)	N/A^c^	N/A
	Stage 5D	6 (55)	10 (56)	N/A	N/A
	Peritoneal dialysis	0 (0)	3 (17)	N/A	N/A
**Fatigued, n (%)**		6 (55)	18 (100)	N/A	N/A
	Stages 4-5	3 (27)	8 (44)	N/A	N/A
	Stage 5D	3 (27)	13 (72)	N/A	N/A
**Sex, n (%)**					
	Male	3 (27)	10 (56)	3 (30)	1 (17)
	Female	8 (73)	8 (44)	7 (70)	5 (83)
**Hospital, n (%)**					
	1	5 (45)	9 (50)	5 (50)	4 (67)
	2	6 (55)	9 (50)	5 (50)	2 (33)
Age (years), mean (SD; range)		65 (14.8; 27-80)	64 (9.9; 45-83)	N/A	N/A
**Profession, n (%)**					
	Nephrologist	N/A	N/A	4 (40)	2 (33)
	Nurse practitioner	N/A	N/A	2 (20)	3 (50)
	(Peritoneal) dialysis nurse	N/A	N/A	2 (20)	1 (17)
	Social workers	N/A	N/A	2 (20)	0 (0)

^a^CKD: chronic kidney disease.

^b^Patients with CKD stages 4 to 5 have advanced kidney disease but do not yet undergo dialysis treatment; patients with CKD stage 5D undergo hemodialysis or peritoneal dialysis treatment.

^c^N/A: not applicable.

### Material

The interviews began with an introduction, and at the prototype phase, patients’ fatigue, their opinions about the rationale of this study (eg, attentional and self-identity bias), a demo of the computer tasks, and the study concept and design were discussed. During the evaluation phase, patients and professionals were asked to evaluate the intervention study, assess the communication with the researcher, and gauge applicability of the training in their medical setting. The translated interview guides for all the interviews can be found in Multimedia Appendix 1.

### Intervention

Both self-identity bias and attentional bias CBM trainings were an adaptation of the original test versions. Instead of the 50%:50% ratio between bias-congruent and incongruent tasks in the IAT and VPT measurements, the training sessions contained 100% bias-incongruent tasks. This means that in the IAT, participants only had to pair “Vitality” together with “Me” and “Fatigue” with “Other.” In the VPT, the target appears only at the same spot as the vitality words. The training sessions took approximately 5 to 10 minutes.

The demo in the prototype phase was created using Inquisit 4 [46]. The computer tasks were adapted based on the feedback received during the prototype phase. For instance, break screens were included to offer participants the option of taking a break during the tasks. During the evaluation phase, the IAT training consisted of 120 trials [27], with 2 break screens (after 40 trials). The VPT training consisted of 102 trials with 4 break screens (after 20 trials). During the evaluation phase, the assessment and training tasks were offered via a combination of Qualtrics software and Gorilla Experiment Builder [47,48]. Owing to the different features in Qualtrics and Gorilla.sc, it was decided to contact, instruct, and ask research questions to the participants via Qualtrics, and for the assessment or training tasks, they were directed to Gorilla.sc.

In our intervention study, the participants first underwent a baseline phase with multiple baseline bias measurements. This was followed by a 2-week training phase with a training session on 6 of the 7 days, combined with 1 bias measurement per week. In the first training week, participants either had IAT training or VPT training. In the second week, they underwent both training paradigms. Then, a 4-week posttraining phase with weekly bias measurements followed.

### Procedure

In this study, 5 patient partners were involved systematically. They contributed to various matters of the project, provided feedback on the information and consent forms, and helped in piloting the interviews. For the interviews in the prototype phase, patients were approached by their own nephrologist or nurse practitioner. During a dialysis check-up or a regular visit to the outpatient department, the care provider gave a brief description of the study and provided the patient with the informed consent form, which was constructed according to Good Clinical Practice regulations.

Most recruited HCPs (recruited equally among the 2 hospitals) were recruiting patients for the study (except for social workers); however, they had limited knowledge of the CBM training used in the study. After sign-up, the researcher contacted the participants to schedule an appointment for the interview at a time and place that was convenient for the participants. For patients, this was scheduled during hemodialysis sessions or at their homes, and for HCPs, this was at their offices. Before the interview, the participants completed a questionnaire regarding their demographic characteristics (Multimedia Appendix 1). The interviews for the prototype phase were conducted between June 2019 and October 2019.

The feedback received in the prototype phase was used in the development of an intervention study to quantitatively evaluate the effectiveness of CBM training. The first participant began the intervention study on January 20, 2020. The intervention study lasted for 8 to 9 weeks. For the interviews in the evaluation phase, patients were informed about the interviews in the information letter for the intervention study. Participants were informed that they could choose to participate in the intervention study only. When participants were in the final weeks of the intervention study, the researcher contacted them to ask whether they were interested in participating in the interviews. The interviews with patients were scheduled after the last measurement for the intervention study (March and April 2020). The professionals in the evaluation phase were all involved in the project, and their interviews took place from April to May 2020. Unfortunately, owing to COVID-19 pandemic restrictions, the interviews in the evaluation phase had to be conducted via a phone call instead of face-to-face. These phone calls were conducted by calling participants via Microsoft Teams on a computer and were recorded via the recording function on a smartphone.

All interviews (Multimedia Appendix 1) were semistructured. The number of participants was based on earlier experiences with similar studies, and for the patients and HCPs at the prototype phase, we believed that data saturation was reached because no new concepts were introduced in the last interviews [49]. During the evaluation phase, all the professionals involved in the intervention study were interviewed. Participants received a small gift after the interview, which was approved by all ethical committees (see the *Ethics Approval* section).

### Data Analyses

The interviews were recorded, transcribed verbatim, coded, and analyzed thematically [50] (see Multimedia Appendix 1 for the code schemes). Atlas.ti and Excel were used to code and analyze the interviews. In the prototype phase, needs and requirements were identified and applied to the intervention study. At the evaluation phase, the intervention study and the adjustments made at the prototype phase were evaluated. In both phases, the acceptability of the training and its applicability to clinical care were assessed.

The codes used in this study were similar to the wording used in the data. In particular, regarding acceptability, the answers were brief; therefore, the results were described more quantitatively. For instance, the code “Did the training help? No” (n=17) has this quote: “I: Do you think the training sessions had influence on you? P: No. I: No? Nothing changed, you did not notice anything? P: No. I: And did you have the feeling that the training sessions helped you? P: No. I: No, okay, so no improvements that you have noticed. P: No,” and adding these quotes did not add much to the interpretation of the data. The data comparing the phases and evaluating the trainings’ applicability in the clinical setting are richer and have more quotes.

### Ethics Approval

We abided by the Ethical Principles of Psychologists and Code of Conduct as set out by the British Association for Behavioural & Cognitive Psychotherapies and British Psychological Society. This study was approved by the Committee of Human Research (*Commissie Mensgebonden Onderzoek*, file number 2019-5816), which decided that legal medical-scientific research with people (*Wet medisch-wetenschappelijk onderzoek met mensen*) did not apply to this research and redirected the study to a local ethics committee. This study was approved by the local ethics committees of the 2 hospitals, Isala and Ziekenhuisgroep Twente, and the University of Twente (file numbers 191020, 19-26, and 191193, respectively).

## Results

### Parameters

This study evaluated the CBM training in patients with CKD and their HCPs. In addition to a general evaluation of the training, acceptability, applicability in the kidney care setting, and the 2 design iterations were explored. Acceptability was evaluated using 7 TFA components [42]. Applicability was assessed by exploring obstacles and possible solutions for implementation in a kidney care setting. Finally, the design process of CBM training was assessed by comparing patients’ and HCPs’ opinions after the first introduction of CBM and after the intervention study.

### General Evaluation of the Training

The training evaluation revealed a 2-faceted picture. On the one hand, at both time points, most patients and professionals were positive about the training. On the other hand, none of the patients reported experiencing benefits from the training. Specifically, none of the patients thought that the training helped them or noticed a positive change during the study. In fact, one patient noticed the opposite effect: she experienced more fatigue during the training weeks. However, 4 patients recognized the implicit or longitudinal nature of the study and indicated that it could still have an effect that they just did not notice yet. Furthermore, many patients still thought that the computer tasks were useful, for instance, making them more aware of their fatigue (n=2); for example, “I: did you think the training helped you in one way or another? P: Yeah,...being a bit more aware about thoughts...when I fill in ‘I am lazy’, or ‘I am vital’, or ‘I am tired’, etcetera, then I think that I should do something about that, what can I do about it, what are causes... I: And did you think the training was useful? P: Yes, it has contributed, yes, especially in the awareness process,” bringing distraction (n=2), confirming capabilities (n=1), and “brain-training” (n=1). Similarly, 61% (11/18) of patients thought the study was fun, indicating, for instance, that they saw it as a way to learn about themselves (n=3), a new way to pass time (n=2), or as a game (n=2). HCPs were positive about the training because it could be helpful for participants (n=4) because the setup is charming and not burdening for patients (n=1) and because it is important to find something that could help against fatigue as it is a frequent symptom in patients (n=1).

Although around a third of the participants (n=7; 39%) complained about the computer tasks, others praised that it was quick and easy. Specifically, they liked that the training took less than 15 minutes (n=14), they did not mind the daily training sessions (n=9), and they thought the training was not difficult (n=3) and that everyone could do them (n=2). The most frequently reported complaint was monotony: 7 participants mentioned 14 times that there was too much repetition and that this made the training boring, especially as the tasks were always the same (mentioned 12 times by 6 participants). Remarkably, only 11% (2/18) of patients had noticed the difference between the measurement and the training sessions (even though this was mentioned in the emails and the instructions), which may have amplified this complaint. Furthermore, although 11 participants thought the study in general was clear, 4 participants mentioned that the purpose of the study was not clear to them, and 9 participants said that they just did whatever the researcher sent them without thinking much about the content. Thus, mixed results were found regarding understanding of and affinity for training.

### Applicability

Most patients and HCPs were positive about applicability, indicated by the willingness of all HCPs and most patients to participate again in a similar study (n=14) and their support for wider implementation of the training (n=13). In total, 14 patients would continue with the training, in its current form (because it could still help, n=3; to help the researchers, n=2; and for evidence, n=1), after confirmation (when evidence is found, n=2), or adjustment (with different frequencies, n=2). In total, 14 patients would also recommend the training to others. Different reasons were mentioned: (1) although it did not work for them, it could still work for others (n=3); (2) because it should be tried out on more people (n=1); and (3) because it is simple (n=1). Others would recommend the training but not to older adults (n=2) or only after more evidence is found (n=2). Not having improved themselves was the biggest reason for not recommending the training to others (n=3).

The most mentioned obstacle for delivering CBM to patients with CKD was patients’ computer skills (mentioned by all professionals during the evaluation phase). The involved HCPs estimated that about 50% to 60% of all patients with CKD were interested in the intervention study but that 40% to 66% of them could not participate because of low digital literacy or the lack of a laptop. The intervention study offered participants the option of borrowing a laptop. However, the 2 participants who borrowed a laptop stopped the intervention study because they lacked the skills to interact with the borrowed laptop, among other reasons.

In contrast to the previous observation, however, the complex procedure with the 2 programs (Qualtrics and Gorilla.sc) in the intervention study, to our surprise, was not reported as an obstacle by the patients. They were positive about the 2 programs (n=17) and the transition (n=9). Furthermore, of the participants who completed the study, 83% (15/18) of patients were able to perform the tasks without assistance. Participants reported that they had to take a good look for the first time, but after that, they knew what to do and had no problems with it. Possible solutions mentioned for the lack of computer skills are providing the training on an app; 4 patients preferred the training on a tablet instead of a computer and 3 HCPs agreed that an app is more accessible. Other recommendations regarded instructions, both for HCPs (n=2), for example, “I would first explain to the specialists, the nurse practitioners and the nurses that it exists and how it works and what patients have to do for it” (HCP 5), and for patients; clear instructions (n=2) by making an instruction video (n=2) or an information leaflet (n=1) and providing it on the web (n=2) or by providing a help desk (n=2).

Interestingly, another obstacle for delivering the training to patients was a discrepancy between the patients’ and the professionals’ views on patients’ fatigue severity (mentioned by 3 professionals):

I was also quite surprised that at first you think that someone is eligible but then indicates that they do not suffer from fatigue and that we were really surprised like, oh, okay, you know, they do give those complaints back but then you really come to the core and then you ask them you know, if they want to be a participant for the study and then, well, they turned out to be not as tired as we had thought they were. Not tired enough to want to participate in this.

HCP 3

Moreover, because of the fluctuating nature and the subjective experience of fatigue, it can be hard for professionals to interpret patients:

Look, in any way, we quickly have an opinion,...but I am also aware that that is not always the truth so to say, there are people that complain bitterly in the moment that they sit in my office but when you bump into them at the mall, then you think, well, actually he functions fine and he is chatting with everyone, he seems to be alright, and the other way around as well, people that say here, “yes doctor, alright doctor, everything is fine,” but next, at home actually do not get off the couch because they are actually not able to anymore, so that is something that I ask about actively but still, people do not always show everything, so that is not, it is a certain impression that I get of that, that does not have to be the truth, you try to get a picture, that is what it comes down to.

HCP 2

One patient proposed adding physical tests to measure fatigue, suggesting a desire for more objective measurements of fatigue and possible improvements. Thus, the fluctuating nature and subjective experience of fatigue impedes communication between HCPs and patients and could also be a barrier for recruitment and adherence to future fatigue interventions.

About the applicability of the training at the nephrology department, 2 professionals mentioned that a clear plan for the integration of the training in regular treatment is important:

I think if you only give it to someone and just let that person go, you don’t do anything with it, then I don’t know if that will work. I think they do need some support,...I think it is good to have someone that they can fall back to and who asks how it is going and whether they have encountered issues.

HCP 4

In addition, professionals thought that dialysis nurses (n=3), social workers (n=1), or family members (n=1) could help. However, one professional mentioned the willingness of nurses as an obstacle and 3 professionals recommended using representatives among the nurses; for example, “at the dialysis hall, there I would already from the top of my head make one or two nurses responsible that they make sure that the conditions at the hall, for instance the laptop etcetera, that that is taken care of” (HCP 5). Thus, for further implementation, it is important to have a clear plan for the introduction of training at the hospital, both regarding the HCPs’ roles and in interaction with already existing treatment offered to patients. The professionals also provided suggestions for other delivery methods, including patient associations (n=2), peer meetings (n=1), and presentations at theme nights (n=1).

### Acceptability

The evaluation of the CBM training provides a mixed picture of acceptability. The generally positive evaluation suggests positive reflections on affective attitude (how participants feel about the intervention), ethicality (whether the intervention has a good fit with the participants’ values), and opportunity costs (whether benefits, costs, or values have to be sacrificed to engage in the intervention). Furthermore, the high number of patients and HCPs recommending or being interested in continuing the training also suggests a positive assessment of opportunity costs. However, as no participant experienced a direct effect from the training, perceived effectiveness (whether the intervention is perceived to be likely to achieve its purpose) was evaluated negatively.

The other components received mixed evaluations. For instance, burden (the amount of effort perceived to be required for intervention participation): as some participants complained about the tasks being repetitive and boring, those participants may have perceived the training as a burden; however, others liked that the tasks were simple and quick. Similarly, although most participants thought the study and its explanation was clear, only 2 of them had understood the difference between the training and measuring tasks and 9 did not think much about the content. Therefore, the picture of intervention coherence (whether the participant understands the intervention and how it works) is mixed. Finally, large individual differences were found regarding self-efficacy (the participant’s confidence in performing the behaviors required for intervention participation), with computer skills being the most mentioned obstacle for current and future recruitment, but also participants being positive and not needing help with the computer tasks.

### Developmental Process

As can be expected, changes in the design that were applied following the prototype stage were confirmed as improvements in the second evaluation after training. However, when comparing the results from both iterations more closely, some surprising inconsistencies were observed. For instance, some suggested changes that were applied after the first phase received negative feedback during the evaluation phase (eg, break screens; n=12) and some that were not applied were not missed during the evaluation phase (eg, sounds, pictures, and colors). Actually, the simplicity of the computer tasks was valued positively (n=17) at the second evaluation; for example, “I think when you’re tired that it is nice that it is very simple and that there are not too many bells and whistles added because you do have a certain tiredness so then that is nice because it is very clear” (Participant 3). Furthermore, video instructions were advised during the prototype phase but were not missed during the evaluation phase. Most participants were explicitly positive about the written instructions. However, 2 professionals again recommended video instructions to improve the applicability of training. In addition, 3 participants mentioned that they would have liked more personal contact with the researcher and suggested calling (with video). Finally, the 2 phases show the difference between expectations after one session and experiences with many sessions. At the prototype stage, concern was expressed toward monotony within sessions. Conversely, in the evaluation phase, participants were positive about the stimuli (n=15) and did not mind repetition within the sessions (n=12).

## Discussion

### Summary of Results

The aim of this study was to carefully design and evaluate a novel CBM training program for patients with CKD by considering their needs and opinions and those of their HCPs. In an iterative design assessed in 2 developmental stages, the training was generally evaluated positively with some minor points for improvement. Acceptability (evaluated using the 7 components of the TFA [42]) revealed a mixed evaluation: effectiveness was not perceived by patients; burden, intervention coherence, and self-efficacy received mixed evaluations; and affective attitude, ethicality, and opportunity costs were evaluated positively. Although applicability in clinical care was evaluated positively, barriers were also encountered, such as patients’ low digital literacy, the subjectiveness of fatigue challenging communication between patients and HCPs, and perceived effectiveness. Furthermore, challenges for applicability in the nephrology department are how the training would be integrated in regular treatment and in what way HCPs would be involved. Possible solutions included assigning representatives among dialysis nurses, offering training on an app, providing clear instructions, and offering assistance via a help desk. Finally, the results showed that the evaluations at the different development stages provided very different opinions that complemented each other, indicating that the iteration was useful for the design process.

### CBM Improvement

The findings regarding the acceptability of the training confirmed the results reported by Beard et al [31]. In both studies, participants were generally positive about CBM training but were also skeptical about its effectiveness and complained about the monotonous nature of repetitive tasks. Laurens et al [19] received similar comments about their alcohol-avoidance CBM app. Even de Voogd et al [51], who included many steps to increase compliance and engagement (eg, a progress bar; feedback; financial compensation; and text, email, and phone reminders) in their study focused on anxiety and depression, still encouraged the investigation of other motivating features. Laurens et al [19] recommended educating users about the rationales behind CBM and its repetitive tasks. Our results support these recommendations: more engaging formats and explanatory content for CBM users are needed. Such an engaging format could be gamification [52]. Promising results have already been found with a virtual reality setup for interpretation bias training countering trait anxiety [53,54] and a virtual reality setup for attentional bias training to counter social anxiety [55].

Another solution for the complaint about monotony of tasks is to simply reduce the frequency of sessions. In our intervention study, participants had to perform at least 21 sessions during the 8- or 9-week study period, with most participants not distinguishing bias measurements from training sessions. Participants in this study suggested voluntary measurement sessions. Similarly, participants could be given more autonomy to personalize their training and decide on their preferred frequency and length of training sessions, which may be assumed to increase acceptance and adherence. However, this may also lead to some patients not achieving the full training potential because CBM is based on the idea that by repeating tasks, implicit associative networks are changed, which can then reflect behavior and cognition. Therefore, repetition is an important aspect of the CBM mechanism. In the context of alcohol-related CBM, Eberl et al [18] estimated the optimal number of CBM sessions to be between 6 and 12. These sessions contained 200 trials [18]; however, especially regarding VPT, the literature varies widely in terms of the number of trials in the sessions [56]. Thus, the optimal CBM dosage, both in terms of the number of sessions and the number of trials within a session, needs further investigation. Similarly, the degrees of freedom that may be given to participants to decide on the dosage are not known.

### Obstacles and Possible Solutions

The obstacles identified in this study are consistent with the results of previous studies on patients with CKD. The found discrepancy between patients’ and professionals’ views on patients’ fatigue severity confirms and underlines the claims by Jhamb et al [57] that fatigue is an underrecognized symptom and that HCPs’ awareness of this symptom should be improved. The authors [57] recommend developing improved methods to define, measure, and screen patients for fatigue to bridge this discrepancy. The training evaluated in this study might be able to provide or facilitate this process.

Furthermore, patients’ low digital literacy was the main reason for their ineligibility in the feasibility study by Hudson et al [58] for a web-based CBT intervention countering psychological distress in patients who underwent hemodialysis. Although patients in the trial by Hudson et al [58] were provided with tablets during hemodialysis, the adherence was low, and 25% of them required brief training in the use of tablets and the internet [58]. In our study, the use of an app was recommended by both patients and professionals. An advantage of apps is that they can be made easily accessible with minimal overt use of the internet. Furthermore, our solution to offer assistance in the form of a help desk might make it more accessible, even for people with limited internet or tablet experience. The effects of these suggestions should be investigated in the next iteration of this training. Furthermore, although the video instructions recommended in the prototype phase were not missed during the evaluation phase of this study, offering both options was also thought to make the training more accessible to patients. Besides, better effects have been reported for multimodal eHealth interventions [34].

### Future Research and Limitations

Our findings regarding the design process support the importance of iterations and ongoing evaluation to ensure successful user-centered design and subsequent clinical implementation of the intervention, as suggested by the CeHRes Roadmap [44] and the Dynamic Sustainability Framework [43]. The opinions at the different stages are valuable, as the expectancies and user experiences show how design elements of the intervention affect both adoption and continued use. The ability to anticipate and repeatedly evaluate patients’ and professionals’ opinions will hopefully enhance the training’s sustainability and success. This study demonstrated that even the use of one iteration adds demonstrably to the quality of the training. Future research should also follow a sustainable intervention design framework.

A limitation of this study was the small sample size. Although we interviewed all professionals directly involved (n=6) in the evaluation phase, the inclusion of a larger sample may have yielded richer data. However, even though the group of HCPs was small, data saturation was reached for most of the topics. Future research should further explore the applicability of training in nephrology settings, as well as other previously formulated suggestions.

### Conclusion

In conclusion, this study evaluated a CBM training countering fatigue by involving patients with CKD and HCPs at 2 different stages of the developmental process: in an early stage of prototyping and after using the training in an intervention study. Overall, the training was evaluated positively, but the acceptability received mixed results. The applicability of the CBM appeared positive, although barriers were identified, such as patients’ low digital literacy and practical integration in the hospitals’ routines. Possible solutions were offered, but further empirical testing is required. By following sustainable intervention design frameworks, this study provided the first steps toward bringing this CBM training countering fatigue to patients. In general, our study clearly shows the necessity of including user perspectives in the development of CBM interventions.

## Appendix

Multimedia Appendix 1 Interview questions, demographics, and code schemes.

## Abbreviations

CBM: cognitive bias modification
CeHRes: Center for eHealth Research and Disease Management
CKD: chronic kidney disease
HCP: health care professional
IAT: implicit association task
TFA: Theoretical Framework of Acceptability
VPT: visual probe task
