# Supplementary material for: Cognitive Bias Modification Training Targeting Fatigue in Patients With Kidney Disease: Usability Study
Source: JMIR Form Res. 2023 May 29;7:e43636. doi: 10.2196/43636 (PMC10262024; doi:10.2196/43636)
Supplement: Multimedia Appendix 1 [file formative_v7i1e43636_app1.docx]

**Appendix A.1: Interview questions Timepoint 1: Patients**

**Introduction**

Thank you for participating in this interview. I am Jody Geerts, junior researcher at the University of Twente. Subsidized by the Kidney Foundation, we are conducting a study on fatigue in renal patients. We want to understand how renal patients experience fatigue. We will also develop a program that we think will help against fatigue. We want this program to suit renal patients. During this interview I am going to ask you questions about being tired and I will show you the program that we are developing. We think that you can give us valuable information. I am especially curious about your ideas and opinions. There are no right or wrong answers, this is about your opinion and why this is your opinion. You are allowed to take breaks if need to. If you don’t mind, the interview will be recorded. The research is anonymous, meaning that your name will not be mentioned in the results of the study. The interview will take about 40 to 60 minutes.

Do you have questions before we start?

*(Before starting the interview, participants sign informed consent if they have not done so before, they answer the demographic questionnaire and the recorder is turned on)*

**Illness and complaints**First I am going to ask you a couple of questions about your illness.

- Which illness(es) do you have?
- How long have you had this/these illness(es)?
- How do the illness(es) influence you?
  - Daily life, well-being, fitness, social relationships

**Fatigue**

- Are you tired sometimes?
- How much do you suffer from being fatigued?
- How do you suffer from being fatigued?
- How would you describe that feeling of tiredness?
  - Are you tired continuously or at certain moments?
- How does being tired influence your life?
  - Can you give examples? (Bv. Daily life, well-being, fitness, social relationships)
- Do you think being tired is something all renal patients suffer from? Can you elaborate?
  - Do you have an idea why there are differences between patients in how much they experience fatigue?

**Coping**

- How do you cope with being tired?
  - Can you give examples?
- Do you have certain habits (e.g. sleeping during dialysis) that you think prevent being tired?
  - What? Why?
  - Do you think those habits work against the fatigue?
  - Do you have ideas how you would like to be helped countering fatigue?
    - E.g. sharing tips with other patients, therapy, courses, etc.

**Fatigue + illness**

- How is in your opinion being tired connected to your illness?
  - Is being tired now different from being tired before you were ill?
    - In what way?
- Do you think your fatigue is influenced by your (dialysis) treatment? Why (not)?
- Did you talk about your fatigue to people in the hospital (e.g., doctors, nurses)?
  - Why (not)?
  - How did that go?
  - What do you think of the help offered to counter fatigue?

**Non-medical treatments**

- Did you ever participate in non-medical treatments or programs to counter your fatigue? E.g., ergotherapy, physiotherapy
- What do you think of these programs?
  - Do you think such a program can decrease fatigue? Why (not)?
  - Are you interested in such a program? Why (not)?
- If you could think of such a program yourself, what would be important? Why?

**Technology**

- Do you have experience using technology (e.g., apps) to support your health? If so, could you describe them?
- What do you think of such technologies?
- Can you imagine that such a program could help renal patients?

**Influence thoughts on fatigue**We think that certain thoughts and habits can enhance fatigue complaints.

- What do you think of this idea?
- Do you recognize yourself in this? Why (not)?
  - Do you have an example?

**Bias**We think that because being tired is so tedious and keeps returning, unnoticed, people can become sensitive to signs of fatigue. People can also start thinking that being tired belongs to them as a person.

In this way people teach themselves subconsciously to give a lot of attention to being tired and seeing it as part of their persona, which only results in experiencing more fatigue.

- Do you maybe recognize yourself in this?
- Can you image that it is possible to unlearn this?
  - How would it be possible to unlearn this, do you think?

**Demo**Soon I will let you do two computer tasks. You can shout out everything that catches your eye, what you think is good, what you think is not good, if you would add something, etc. Everything that comes to mind is welcome. After that, I will ask you some more questions.

**General questions**

- What do you think of these computer tasks?
- Do you think these computer tasks could help you with being tired? Why (not)?
- Would you want to change something about the computer tasks? What? Why?
  - Would you want to add or remove something?
- Would you want to do these computer tasks yourself? Why (not)?
  - Would you recommend these computer tasks to others? Why (not)?
- Do you think the computer tasks are clear, accessible, attractive, understandable, easy to use, fun?
  - What do you think is the most important aspect of the computer tasks?

**More specific questions**

- What do you think of the use of language in the program?
- What do you think of the instructions in the demo?
- Would you change something in the instructions?
- What do you think of the words (stimuli) on the screen?
  - Are these words related to being tired for you? Why (not)?
  - Do you have suggestions for words that we could use?
- One computer tasks had a white background, the other had a black background. Do you have a preference?

**Measurement / Training** **scan per interview how extensive this explanation has to be*
We want to use the computer tasks that you have just seen for both measurements and training sessions. The tasks will be the same for measurements and training sessions, the only difference is that the balance is different.

Dot probe: In the training sessions the E and I that you had to respond to will always be where vitality was. We think that patients have taught themselves to automatically direct their attention to fatigue. By putting the E and I at vitality in the training sessions, we hope that we can reverse this.

IAT: In the measurements in the example you have seen both “Me and Tired” as well as “Me and Vital” together. We think that the combination “Me and Vital” is more difficult for patients because we think that they see themselves as a ‘tired person’. In the training sessions patients will only practice the combination “Me and Vital” to weaken the connection between the self and being tired and to enhance the connection between the self and vitality.

- What do you think of these ideas?
- How do you think patients will react to this explanation?
- Do you think patients will benefit from this?
- Would you want this explanation during the study?
  - Would you for instance want to know when you are doing a training / measurement?

**Study design** **Show the example calendar*
Our idea is that patients will do these computer tasks during a period of 6 to 7 weeks. For half of the patients it will be 6 weeks, for the other half it will be 7 weeks. One group will have 1 week of measurements, and the other will have 2 weeks of measurements before the training sessions will start. The measurements will be take about 10 to 15 minutes and patients will be asked to do the measurements 3 times a week. After those 1 or 2 weeks measurements the daily training sessions will begin. These will take 2 weeks for everyone. In the first week all patients will be trained with one of the computer tasks, in the second week all patients will be trained with both computer tasks. In the first week the training sessions will take about 5 minutes, in the second week the training sessions will take about 10 to 15 minutes. During the training weeks patients will also be asked to do a measurement twice a week. After the two training weeks the participants will be asked to do weekly measurements four more times.

- What do you think of these ideas?
- Would you want to participate in this study? Why (not)?
- How would you like to see this study?
  - Would you want to change something in our ideas?
- Do you think that our ideas are suitable for pre-dialysis and haemodialysis patients? Why (not)?

The idea is to let patients train daily for a period of 2 weeks, about 5 to 10 minutes a day.

- What would you think of this?
- Would you want to change this? Why? (Suggestions?)
- Do you think this is achievable for renal patients?

In total this study will take 6 to 7 weeks.

- What do you think of this?
- Would you want to change this? (Suggestions?)
- Do you think this is achievable for renal patients?

**Implementation daily life**

- Do you think it is easy for you to add this program to your daily life? Why (not)?
  - Suggestions for changes?
- When would be a good moment for you to do this program?
  - For dialysis patients: is this program suitable to do during dialysis? Why (not)?

**Study 2**

In November we would like to start this research.

- How would you preferably like to see this research?
  - Via the computer / via an app
  - With personal contact with the researcher
    - Online / face-to-face?
  - What do you think is important?
- Do you want health care professionals also to play a part in using the app?
  - (e.g., explaining the app, discussing progress with the patient, possibly monitoring results)
  - With which health care professional would you want to discuss this? (E.g., nurse?)

**Contact**If you would like to participate in the study, you would have to do these computer tasks a couple of times during the week.

- What form of contact would you find the most pleasant for this? E.g., whatsapp, sms, e-mail.

Next to that we want to send reminders to patients that did not do the tasks for, for instance, a couple of days.

- What do you think of this?
- How often would you want to get a reminder?
- How would you want to get a reminder?

**Closure**

- We have discussed a lot, maybe there are still some things that you would like to mention? There is room for that now.
- Would you be interested in joining the study that we discussed? With the answers that you have given me, we will adapt the tasks and we hope that we can start the study in November.

*Thank the participant (with the present) and say goodbye.*

**Appendix A.2: Interview questions Timepoint 1: Health care professionals**

**Introduction**Thank you for participating in this interview. I am Jody Geerts, junior researcher at the University of Twente. Subsidized by the Kidney Foundation, we are conducting a study on fatigue in renal patients. We want to understand how renal patients experience fatigue. We will also develop a program that we think will help against fatigue. We want this program to suit renal patients. During this interview I am going to ask you questions about fatigue in patients and I will show you the program that we are developing. We think that you can give us valuable information. I am especially curious about your ideas and opinions. There are no right or wrong answers, this is about your opinion and why this is your opinion. If you don’t mind, the interview will be recorded. The research is anonymous, meaning that your name will not be mentioned in the results of the study, at most your profession will be mentioned. Because of that there is a small chance that it can be traced back. The interview will take about 40 to 60 minutes.

Do you have questions before we start?

*(Before starting the interview, participants sign informed consent if they have not done so before, they answer the demographic questionnaire and the recorder is turned on)*

**Profession**

- Could you briefly describe your profession? What does it entail?
- How long have you been working as this profession?

**Fatigue**

- Can you tell me about the experience of fatigue in patients?
  - How do patients experience fatigue? E.g., dreariness
- Can you tell me about the start and course of fatigue complaints?
  - How is in your opinion the experience of fatigue connected to the treatment of renal patients?
- Are there differences between patients in the experience of fatigue? If yes, why is that, do you think?
- Can you tell me more about the treatment of fatigue in renal patients?
- Do you have an idea of how patients cope with their fatigue themselves? What do you think of how patients cope with fatigue themselves?

**Non-medical interventions**

- Do you offer non-medical interventions to renal patients against fatigue?
  - What do you think of these interventions?
- Do you have an idea what an intervention tackling fatigue should look like? (If you could design an intervention yourself, what would be important?)

**eHealth**

- Do you have experience with eHealth?
  - With eHealth I mostly mean technical additions to regular care, such as apps, chats, forums, etc.?
  - How do you have experience with this?
- What do you think of eHealth?
- Do you think eHealth is appropriate for renal patients? Why (not)?
- Do you think eHealth could contribute to reducing fatigue complaints? Why (not)?

**Influence thoughts on fatigue**We think that certain thoughts and habits can exacerbate fatigue complaints.

- What do you think of this idea?
- Do you recognize this in patients? Why (not)?
  - Do you have an example of this?

**Bias**Research shows that because being tired is so tedious and keeps returning, unnoticed, people can become sensitive to signs of fatigue. People can also start thinking that being tired belongs to them as a person.

In this way people teach themselves subconsciously to give a lot of attention to being tired and seeing it as part of their persona, which only results in experiencing more fatigue.

- What do you think of this idea?
- Do you recognize this in patients? Why (not)?
  - Do you have an example of this?
- How do you think patients will react to this idea?

**Demo**
Soon I will let you do two computer tasks. You can shout out everything that catches your eye, what you think is good, what you think is not good, if you would add something, etc. Everything that comes to mind is welcome. After that, I will ask you some more questions.

**General questions**

- What do you think of these computer tasks?
- Do you think these computer tasks could help patients with their fatigue? Why (not)?
- Would you want to change something about the computer tasks? What? Why?
  - Would you want to add or remove something?
- Would you recommend these computer tasks to patients? Why (not)?
- Do you think the computer tasks are clear, accessible, attractive, understandable, easy to use, fun?
  - What do you think is the most important aspect of the computer tasks?

**More specific questions**

- What do you think of the use of language in the program?
  - Is the use of language appropriate for renal patients?
- What do you think of the instructions in the demo?
- Would you change something in the instructions?
- What do you think of the words (stimuli) on the screen?
  - Are these words related to being tired for you? Why (not)?
  - Do you have suggestions for words that we could use?
- One computer task had a white background, the other had a black background. Do you have a preference?

**Measurement / Training** **scan per interview how extensive this explanation has to be*
We want to use the computer tasks that you have just seen for both measurements and training sessions. The tasks will be the same for measurements and training sessions, the only difference is that the balance is different.

Dot probe: In the training sessions the E and I that you had to respond to will always be where vitality was. We think that patients have taught themselves to automatically direct their attention to fatigue. By putting the E and I at vitality in the training sessions, we hope that we can reverse this.

IAT: In the measurements in the example you have seen both “Me and Tired” as well as “Me and Vital” together. We think that the combination “Me and Vital” is more difficult for patients because we think that they see themselves as a ‘tired person’. In the training sessions patients will only practice the combination “Me and Vital” to weaken the connection between the self and being tired and to enhance the connection between the self and vitality.

- What do you think of these ideas?
- How do you think patients will react to this explanation?
- Do you think patients will benefit from this?
- Would you add this explanation to the study?
  - For instance, do you think patients will want to know when they are doing a training / measurement?

**Study design** **Show the example calendar*
Our idea is that patients will do these computer tasks during a period of 6 to 7 weeks. For half of the patients it will be 6 weeks, for the other half it will be 7 weeks. One group will have 1 week of measurements, and the other will have 2 weeks of measurements before the training sessions will start. The measurements will be take about 10 to 15 minutes and patients will be asked to do the measurements 3 times a week. After those 1 or 2 weeks measurements the daily training sessions will begin. These will take 2 weeks for everyone. In the first week all patients will be trained with one of the computer tasks, in the second week all patients will be trained with both computer tasks. In the first week the training sessions will take about 5 minutes, in the second week the training sessions will take about 10 to 15 minutes. During the training weeks patients will also be asked to do a measurement twice a week. After the two training weeks the participants will be asked to do weekly measurements four more times.

- What do you think of these ideas?
- How would you like to see this study?
  - Would you want to change something in our ideas?
- Do you think that our ideas are suitable for pre-dialysis and haemodialysis patients? Why (not)?

The idea is to let patients train daily for a period of 2 weeks, about 5 to 10 minutes a day.

- What do you think of this?
- Would you want to change this? Why? (Suggestions?)
- Do you think this is achievable for renal patients?

In total this study will take 6 to 7 weeks.

- What do you think of this?
- Would you want to change this? (Suggestions?)
- Do you think this is achievable for renal patients?

**Implementation daily life**

- Do you think it will be easy for renal patients to add this program to their daily life? Why (not)?
  - Suggestions for changes?
- When would be a good moment for patients to do this program do you think?
  - For dialysis patients: is this program suitable to do during dialysis? Why (not)?

**Study 2**In November we would like to start this research.

- How would you preferably like to see this research? How do you think this study could be offered best to renal patients? Why?
  - Via the computer / via an app
  - With personal contact with the researcher?
    - Online / face-to-face?
  - What do you think is important for this?
- Do you want health care professionals to play a part in using the app?
  - (e.g., explaining the app, discussing progress with the patient, possibly monitoring results)
  - Which health care professional would be best for this? (E.g., nurse?)

**Contact**If patients would like to participate in the study, they would have to do these computer tasks a couple of times during the week. We want to send these tasks to participants in the form of weblinks in emails.

- What form of contact would you find best for this? E.g., WhatsApp, sms, e-mail.

Next to that we want to send reminders to patients that did not do the tasks for, for instance, a couple of days.

- What do you think of this?
- How often would think is appropriate to send a reminder to patients?
- How would you send a reminder to patients?

**Closure**

- We have discussed a lot, maybe there are still some things that you would like to mention? There is room for that now.

*Thank the participant (with the present) and say goodbye.*

**Appendix A.3: Interview questions Timepoint 2: Patients**

**Introduction**Thank you for participating in this interview. The past weeks you have participated in the research project VitalME about fatigue in renal patients. We are very curious about how you have experienced that study. In this interview I will ask you questions about various aspects of that study. It is important that you answer honestly, also when you have had negative experiences, because we are doing this interview to improve the study. So, there are no right or wrong answers, it is purely about your experience and your opinion. You are allowed to take a break, just let me know if you want to take a break. If you don’t mind, the interview will be recorded so that I can write it all down later. The study is anonymous, your name will not be used in the results of the study. The interview will take about half an hour to an hour.

Do you have questions before we start?

**Research**

- What did you think of this study? How did you experience this study?
  - Did you think this study was clear? Why (not)?
  - Did you think this study was fun? Why (not)?
  - Do you have improvements for this study?
- What did you think of the length of this study (6-7 weeks + a last post-measure)?
- What did you think of the explanation of this study?

**E-mail contact**

- What did you think of the e-mail system?
- Do you have improvements / suggestions for the e-mail system?
- Do you recommend another way of communicating? Why (not)?
- Would you participate in this kind of study more often?
- What did you think of the way of contact in the e-mails?
  - What did you think of the text in the e-mails?
  - What did you think of the tone in the e-mails?
- What did you think of the number of e-mails?
- What did you think of the varying frequencies of the e-mails?
  - In the first weeks it was 3 emails per week, then it was 6 and then 1 email per week. Were those changes a surprise?
  - Did you find it hard to keep up with the schedule?
    - Why was that?
- What did you think of those weeks that you had to do the study 6 days a week?
  - Did you think that was too much?
  - Some participants were unsuccessful doing the training 6 times a week. Why do you think they were unsuccessful?
    - Is there something we could do about that?
- Did you receive reminders?
  - What did you think of the reminders?
- Did you receive thank-you e-mails?
  - What did you think of the thank-you e-mails?

**Computer programs**

- What did you think of the computer programs in which you did the computer tasks and the questionnaires?
- What did you think of the appearance of the computer programs? (colours, name, lay-out, etc.)
  - The computer tasks were kept simple on purpose. What do you think of this?
  - Do you have improvements / suggestions for the computer programs?
- What did you think of the instruction text in the programs?
  - Did you think the instructions were clear?
  - Did you understand what you had to do when you did the computer tasks for the first time?
  - Do you have improvements / suggestions for the instructions?
- I saw that the computer tasks sometimes went wrong for some participants. Do you have an idea what could have gotten wrong?
  - Did you have something you had difficulty with?
- The explanation of the computer tasks was entirely via the computer programs. Would you have preferred a different way of explaining the tasks? (E.g., face-to-face by a nurse / the doctor / the researcher)
- What did you think of the transition from the first computer program to the other computer program?

**Computer tasks**

- What did you think of the computer tasks (in Gorilla)?
- Do you have improvements / suggestions for the computer tasks?
- In the e-mails it said that the computer tasks would take about 15 minutes. Was this accurate for you?
  - What did you think of this duration?
- Do you think the duration of the computer tasks have an influence on your motivation for the study?
- Did the words in the computer tasks (stimuli) address vitality and fatigue in your opinion?
  - Why (not)?
- There was quite a lot of repetition in the tasks. What did you think of that?
  - Would you have preferred more variety?

**Training**

- What did you think of the training sessions? / How did you experience the training sessions?
- Do you think the training sessions had an influence on you?
  (did you notice anything different, did anything stand out, did anything change?)
  - Did you think the training sessions helped you?
    - Why did the training session (not) help you? How do you feel about this?
- Did you think the training sessions were useful? Why?
  - Was it clear why the training sessions could have been useful? / How the training sessions could have helped you?
- Did you like the training sessions? Why?
- Do you have improvements / suggestions for the training sessions?
- What did you think of the difference between training sessions and measurements?
  - Was this difference important for you?
- What did you think of training week 1 and training week 2?

**Dialysis & Health care professionals**

- Did you do the study during dialysis?
  - Why (not)?
- Did you talk to your doctor or nurses about this study?
  - Why? How was this talk?
  - Would you want to see this differently? E.g., would you want more interest / involvement from the doctors / nurses?

**Family & friends**

- Did you talk to people around you about this study?
  - What was their reaction?
  - Did people around you help with the study? (E.g., the computer tasks, the computer, help with reminders for the measurements / training sessions)
    - Why did you (not) need this help?

**Implementation**This study is the beginning to develop a training that could help against fatigue. If it is the case that the training indeed helps against fatigue and it has received positive reactions, we are hoping that we can develop the training in a way that it will stay available for patients.

- What do you think of that?
- Would you want to continue doing the training? Why (not)?
- Would you recommend the training to others? Why (not)?

**Closure**

- We talked about a lot of different topics. Do you have any further comments or anything else you would like to share?

Thank you + present

**Appendix A.4: Interview questions Timepoint 2: Health care professionals**

Thank you for participating in this interview. The past weeks your patients have participated in the research project VitalME about fatigue in renal patients. We think your opinion and experiences are also very useful for us, that’s why we also wanted to do this quick interview with you. It is important that you answer honestly, also when you have had negative experiences. If you don’t mind, the interview will be recorded so that I can type it all out later. The study is anonymous, your name will not be used in the results of the study. The interview will take about 15 minutes.

Do you have questions before we start?

**The study in general**

1. It has been a while since you have been involved in this study. Is the content of the study still clear to you?
2. What has been your role in this study?
3. What did you think of this study?
4. What did you think of the idea behind this study? *(computer tasks that can help against fatigue)*
   1. What did you think of the intervention / training sessions?
   2. What did you think of the measurements / the research design?
5. Would you participate in this study again? Why (not)?
6. Did you talk to colleagues about this study?
7. Do you have improvements for this study?

**Recruitment**

1. How did the recruitment go in your opinion?
   1. Did you think the recruitment was a burden?
2. Was it hard to find patients?
3. Do you have improvements for the recruitment for this study?
4. Did you talk to patients (dialysis / predialysis) about this study?

If yes, what were their reactions?

**Implementation**
If this study will result in positive results and the training is shown to help against fatigue, we would like to continue developing the training so that it stays available for patients.

1. What would you think of that?
2. How would you envision implementation of this training? / How would you set about implementation of this training?
   1. What would be your role?
   2. Do you see obstacles or opportunities for implementation of this training?
      1. How could those obstacles be solved?
3. How can this training best be offered at a department like nephrology?
4. For who do you think this training is suitable?
   1. Is there a specific group of patients for whom this training would be suitable or is it versatile?
      1. Why?

**Closure**

1. Do you want to mention anything else?

Thank you for this conversation.

**Appendix B: Demographic questionnaire**

Date: Participant number:

Before we start the interview, I would like to ask you to answer the questions below.

What is your gender?

- Male
- Female
- Other

What is your age?

What is your ethnicity?

- Western
- Asian
- Arabic
- African
- Other, namely:

Appendix C.1: Code scheme Timepoint 1 - Patients and Health care professionals

| **First level code** | **Second level code** | **Fourth level code** | **Fifth level code** | **Sixth level code** | **n (quotes)** | **n (pp)** |
| --- | --- | --- | --- | --- | --- | --- |
| Computer tasks: additions / adaptations | | |  |  | 48 | 19 |
|  | no improvements |  |  |  | 6 | 6 |
|  | Contact with patients | |  |  |  |  |
|  |  | thank you present |  |  | 1 | 1 |
|  |  | reward, well done |  |  | 1 | 1 |
|  |  | involve family |  |  | 1 | 1 |
|  |  | accessible |  |  | 1 | 1 |
|  |  | explanation importance of tasks | |  | 4 | 3 |
|  | Organisational |  |  |  |  |  |
|  |  | reward: well done |  |  | 1 | 1 |
|  |  | more compact |  |  | 1 | 1 |
|  |  | dialysis screens |  |  | 1 | 1 |
|  |  | involve family |  |  | 1 | 1 |
|  |  | bigger screen |  |  | 1 | 1 |
|  |  | later post-test |  |  | 1 | 1 |
|  |  | physical test |  |  | 1 | 1 |
|  |  | not on Sunday |  |  | 2 | 2 |
|  |  | optional measurements? | |  | 1 | 1 |
|  |  | Idea for patient recruitment | |  | 1 | 1 |
|  |  | time indication |  |  | 1 | 1 |
|  | Tasks |  |  |  |  |  |
|  |  | more compact |  |  | 1 | 1 |
|  |  | repetition stimuli |  |  | 3 | 2 |
|  |  | Instructions |  |  | 1 | 1 |
|  |  |  | clear transition practice - test | | 1 | 1 |
|  |  |  | clear, concise instructions | | 1 | 1 |
|  |  |  | end screen |  | 1 | 1 |
|  |  |  | IAT about yourself? | | 2 | 1 |
|  |  |  | instructions not too long | | 2 | 2 |
|  |  |  | verbal instructions | | 4 | 3 |
|  |  |  | as fast and as flawless as possible | | 1 | 1 |
|  |  | Game element |  |  |  |  |
|  |  |  | scores |  | 2 | 2 |
|  |  |  | game |  | 2 | 1 |
|  |  |  | against each other | | 2 | 1 |
|  |  | Length of time |  |  |  |  |
|  |  |  | slower |  | 2 | 2 |
|  |  |  |  | words @ dot-pro | 14 | 9 |
|  |  |  | can be longer |  | 1 | 1 |
|  |  |  | not too long |  | 1 | 1 |
|  |  | Additions |  |  |  |  |
|  |  |  | sound |  | 1 | 1 |
|  |  |  | bigger letters |  | 3 | 2 |
|  |  |  | buttons |  | 7 | 4 |
|  |  |  | break |  | 2 | 2 |
|  |  |  | visual |  |  |  |
|  |  |  |  | colours | 2 | 2 |

| icons | 3 | 2 |
| --- | --- | --- |
| explain why it ha | 1 | 1 |
| questionnaire | 1 | 1 |
| Background |  |  |
| no preference | 11 | 11 |
| preference white | 6 | 6 |
| preference black | 2 | 2 |
| Computer tasks: initial opinion |  |  |
| understandable etc. | 31 | 21 |
| first reaction | 23 | 18 |
| instructions | 26 | 19 |
| cross at error | 2 | 2 |
| clear | 1 | 1 |
| transition practice - test clearer | 1 | 1 |
| break | 1 | 1 |
| opinion | 6 | 2 |
| stimuli | 29 | 20 |
| certain words problem | 5 | 5 |
| good / no comments | 15 | 12 |
| language use | 20 | 18 |
| during tasks | 18 | 17 |
| no verbal explanation | 5 | 5 |
| verbal explanation necessary | 6 | 6 |
| start again | 2 | 2 |
| recognizable | 14 | 12 |
| Too confronting | 1 | 1 |
| tiring | 3 | 3 |
| Contact with patients | 6 | 4 |
| thank you present | 1 | 1 |
| involve family | 1 | 1 |
| introduce the intervention well | 23 | 10 |
| Coping |  |  |
| stay active | 10 | 5 |
| possible to unlearn | 3 | 3 |
| distraction | 13 | 5 |
| other classification of life | 5 | 3 |
| experienced worse | 1 | 1 |
| talk about it | 1 | 1 |
| family | 1 | 1 |
| habit | 2 | 1 |
| healthy life | 2 | 2 |
| it belongs to me | 1 | 1 |
| I'm bummed about it | 1 | 1 |
| I'm taking it a bit slower | 2 | 2 |
| ik don't do much | 3 | 3 |
| ik don’t want to be confronted with it | 1 | 1 |
| influence partner | 6 | 4 |
| you have to persevere | 6 | 4 |
| you have to fight it | 2 | 2 |
| you have to accept that you have to live with it | 12 | 7 |


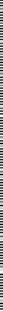

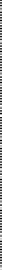

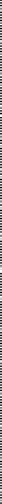

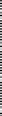

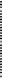

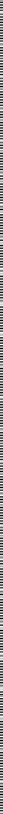

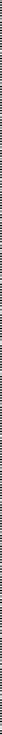

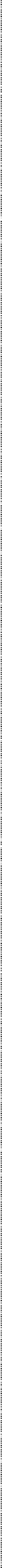

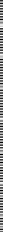

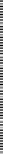

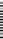

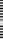

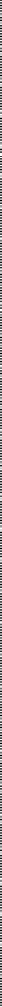

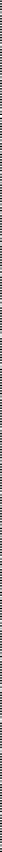

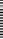


| complain | 1 | 1 |
| --- | --- | --- |
| undertake less | 4 | 3 |
| possibilities instead of limitations | 12 | 6 |
| positive view | 15 | 4 |
| rest / seek relaxation | 7 | 5 |
| sleep | 6 | 5 |
| sport / hobbies | 5 | 3 |
| drink a lot | 1 | 1 |
| what do others think | 2 | 2 |
| sit | 2 | 1 |
| stopped exercising | 1 | 1 |
| Coping? Passive | 5 | 2 |
| Depression + mourning | 1 | 1 |
| Dialysis |  |  |
| Dialysis also has advantages | 1 | 1 |
| decline other dialysis patients | 6 | 4 |
| Dialysis = restrictive | 5 | 4 |
| dialysis helps against fatigue | 7 | 5 |
| dialysis hangover | 4 | 3 |
| heavy treatment | 5 | 5 |
| intervention at dialysis | 39 | 16 |
| dialysis screens | 1 | 1 |
| bigger screen | 1 | 1 |
| planning | 5 | 4 |
| change sleeping pattern | 1 | 1 |
| sleep | 1 | 1 |
| lessened autonomy | 1 | 1 |
| fatigue at dialysis | 10 | 9 |
| fatigue explained | 2 | 2 |
| difference long time dialysis | 3 | 3 |
| room has influence | 2 | 2 |
| eHealth |  |  |
| can it help? | 14 | 11 |
| not-medical interventions | 38 | 21 |
| technology | 31 | 20 |
| Instructions |  |  |
| clear | 1 | 1 |
| transition practice - test clearer | 1 | 1 |
| break | 1 | 1 |
| Intervention |  |  |
| goal | 2 | 1 |
| own idea for intervention |  |  |
| adjustments existing: |  |  |
| other tasks | 1 | 1 |
| cycling with VR glasses | 1 | 1 |
| more dialysis | 1 | 1 |
| activate | 1 | 1 |
| activities | 2 | 2 |
| activating coach | 4 | 3 |
| fysiotherapy | 2 | 2 |


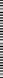

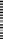

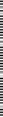

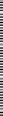

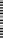

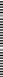

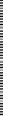

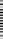

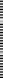

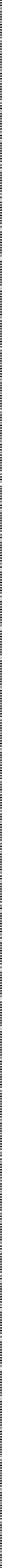

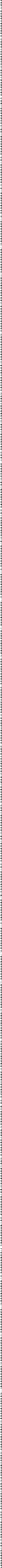

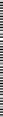

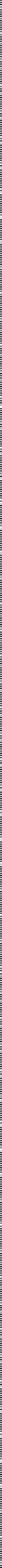

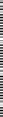

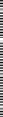

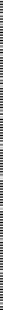

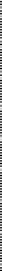

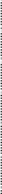


| 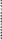 empowerment | 1 | 1 |
| --- | --- | --- |
| rest - exercise scheme | 4 | 4 |
| relaxation exerci | 1 | 1 |
| advice + fellow sufferers |  |  |
| advice | 2 | 2 |
| contact with fellow sufferers | 2 | 2 |
| game | 1 | 1 |
| scores | 1 | 1 |
| game op tv screens | 1 | 1 |
| no own idea for intervention | 7 | 7 |
| change thoughts | 12 | 11 |
| fit? | 1 | 1 |
| yes | 9 | 5 |
| can it help? | 41 | 19 |
| opinion idea | 32 | 18 |
| reaction patients idea intervention | 8 | 7 |
| reaction explanation | 12 | 9 |
| add to daily life | 23 | 17 |
| explanation measurement / training | 38 | 21 |
| want to do more often | 6 | 5 |
| want to do the intervention at home | 5 | 5 |
| no complaints myself | 1 | 1 |
| Reminders |  |  |
| Reminders + importance research | 1 | 1 |
| good | 13 | 13 |
| not necessary | 2 | 2 |
| via mail | 4 | 4 |
| via sms | 7 | 6 |
| Calender / Planning |  |  |
| not on Sunday | 2 | 2 |
| optional measurements? | 1 | 1 |
| no pronounced preference | 2 | 2 |
| good this way | 21 | 17 |
| intensive | 4 | 3 |
| take holidays into account | 3 | 3 |
| vacation | 1 | 1 |
| not for me, for someone else | 1 | 1 |
| possible objections: time + confronting | 1 | 1 |
| Laptop / tablet / telephone | 1 | 1 |
| coordinate with patient | 6 | 6 |
| Contact with researcher | 10 | 9 |
| personal | 22 | 8 |
| mail / sms / telephone | 32 | 20 |
| other option: screens at dialysis | 1 | 1 |
| preference computer | 8 | 7 |
| computer with good instructions | 3 | 3 |
| preference laptop | 1 | 1 |
| preference tablet | 11 | 11 |
| preference telephone | 4 | 4 |
| take it yourself | 1 | 1 |


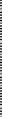

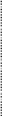

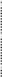

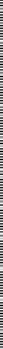

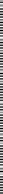

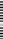

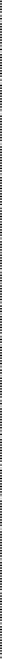

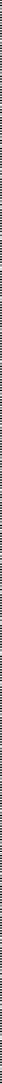

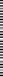

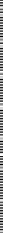

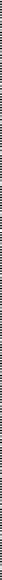

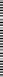

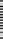

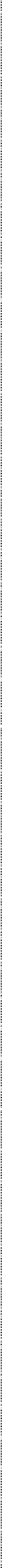

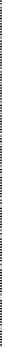

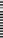

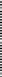

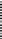

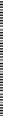

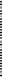

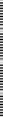

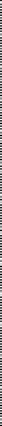

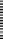

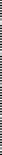


|  | borrow laptop |  |  |  | 3 | 3 |
| --- | --- | --- | --- | --- | --- | --- |
| Patients |  |  |  |  |  |  |
|  | difference kidney patients | |  |  | 31 | 14 |
|  | condition |  |  |  | 15 | 8 |
|  | negative aspects of illness | |  |  | 6 | 2 |
|  | illness / illnesses |  |  |  | 23 | 10 |
|  | patient group |  |  |  | 16 | 9 |
|  | body holds back |  |  |  | 12 | 7 |
|  | not tired |  |  |  | 12 | 4 |
|  | how tired? |  |  |  | 15 | 6 |
|  | predialysis |  |  |  | 6 | 5 |
|  | problems because of illness | |  |  | 1 | 1 |
|  | limitations |  |  |  | 1 | 1 |
| motivation patients | |  |  |  | 2 | 2 |
| Fatigue | treatment fatigue |  |  |  | 3 | 2 |
|  | varying fatigue |  |  |  | 1 | 1 |
|  | fatigue worst symptom | |  |  | 2 | 2 |
|  | negative impact fatigue | |  |  | 1 | 1 |
|  | medical explanations | |  |  | 21 | 14 |
|  | when tired |  |  |  | 1 | 1 |
| Exemplary function | |  |  |  | 3 | 1 |
| Health care professionals | |  |  |  |  |  |
|  | interaction patient - doctor | |  |  | 12 | 5 |
|  | role health care professionals | |  |  | 26 | 20 |
|  | talk about fatigue |  |  |  | 8 | 8 |
|  | difference back in the day | |  |  | 6 | 4 |
|  | misunderstanding practitioners | |  |  | 2 | 1 |
|  | professional: function | |  |  | 10 | 10 |

Appendix C.2: Code scheme Timepoint 2 - Patients

| **Codes** | **Second level code** | **Third level code** | **Fourth level code** | **Fifth level code** | **N Quotes** | **N PP** |
| --- | --- | --- | --- | --- | --- | --- |
| Evaluation training sessions | Training useful? |  |  |  |  |  |
| Usefulness |  | Did the training help? No | |  | 26 | 17 |
|  |  |  | Training actually tiring | | 4 | 1 |
|  |  | Training useful: No | |  | 3 | 3 |
|  |  |  | but maybe it's me |  | 3 | 2 |
|  |  | Training useful: Yes | |  | 1 | 1 |
|  |  |  | awareness / you start thinking about it | | 4 | 2 |
|  |  |  | distraction |  | 2 | 2 |
|  |  |  | confirmation of one's own abilities | | 3 | 1 |
|  |  |  | brain training |  | 1 | 1 |
|  |  | Training useful: don't know, maybe effect | | | 5 | 4 |
| Evaluation training sessions | Computertasks |  |  |  |  |  |
| General |  | Computertasks: length | |  |  |  |
|  |  |  | Quarter max. necessary? Yes | | 21 | 17 |
|  |  |  | Quarter length fine |  | 14 | 14 |
|  |  |  | Length & motivation: doesn't matter | | 5 | 5 |
|  |  |  | Rather not longer |  | 5 | 4 |
|  |  |  | Length & motivation: nice that it's short | | 2 | 2 |
|  |  | opinion positive |  |  |  |  |
|  |  |  | Stimuli |  | 15 | 15 |
|  |  |  |  | fine | 15 | 15 |
|  |  |  |  | repetition stimuli doesn’t matter | 12 | 12 |
|  |  |  | fine |  | 10 | 7 |
|  |  |  | not difficult |  | 5 | 3 |
|  |  |  | Repetitition computertasks: not annoying | | 3 | 3 |
|  |  |  | no improvements |  | 2 | 2 |
|  |  |  | good to do for everyone | | 2 | 2 |
|  |  | opinion negative |  |  |  |  |
|  |  |  | repetition computertasks: a lot + annoying | | 14 | 7 |
|  |  |  | always the same |  | 12 | 6 |

|  | nicer with more variety | 4 | 3 |
| --- | --- | --- | --- |
|  | no challenge | 2 | 2 |
|  | not useful | 2 | 1 |
|  | not fun | 1 | 1 |
|  | Computertasks & fatigue |  |  |
|  | actually tiring | 4 | 2 |
|  | sore arm | 1 | 1 |
|  | more fatigue, less concentration, more mistakes | 1 | 1 |
|  | Computertasks: Make mistakes |  |  |
|  | Mistake due to expectation | 4 | 4 |
|  | Be alert for change | 3 | 3 |
|  | Distracted by surroundings | 3 | 3 |
|  | Mistake due to eye-hand coordination | 2 | 2 |
|  | Mistake due to answering too quickly | 2 | 2 |
|  | Mistake due to waiting for dot | 2 | 1 |
|  | Mistake due to small keyboard | 1 | 1 |
|  | More mistakes at certain time of day | 1 | 1 |
|  | IAT & VPT |  |  |
|  | IAT was fun, VPT was boring | 4 | 2 |
|  | Computertaasks: IAT pay attention | 2 | 2 |
|  | Computertasks: VPT |  |  |
|  | VPT don't look at words | 6 | 4 |
|  | VPT distracted by words | 1 | 1 |
|  | VPT simple | 1 | 1 |
|  | VPT didn't understand why words | 1 | 1 |
|  | Computertasks: faltered sometimes | 3 | 3 |
| Evaluation study | Research general |  |  |
|  | Research: positive |  |  |
|  | participate more often: yes | 15 | 14 |
|  | clear / fine: yes | 14 | 11 |
|  | improvements? No | 12 | 11 |
|  | Mailing system / contact: good | 36 | 17 |
|  | Amount of emails: good | 16 | 14 |


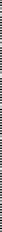

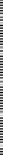

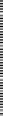

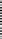

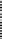

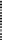

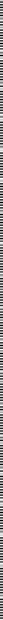

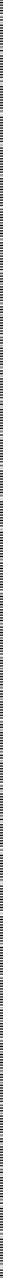

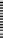

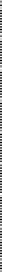

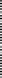

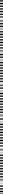

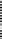


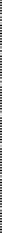

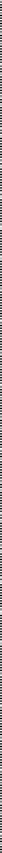

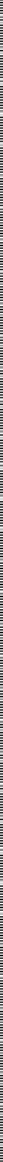

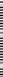

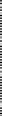

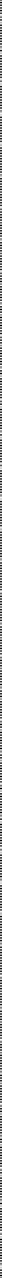


| Thank you emails |  |  |
| --- | --- | --- |
| neat / fine / alright | 17 | 16 |
| not necessary | 2 | 2 |
| didn't get them | 1 | 1 |
| Reminders |  |  |
| fine | 15 | 15 |
| other way communicating: No | 11 | 10 |
| No, at ease at home | 3 | 3 |
| Research schedule |  |  |
| training daily: no problem | 12 | 9 |
| varying schedule: fine | 10 | 10 |
| you send, I follow | 10 | 9 |
| schedule: intense, but not too muc | 4 | 4 |
| schedule clear | 2 | 2 |
| Not intense | 1 | 1 |
| participate when it works for me | 1 | 1 |
| handy that everything came at the | 1 | 1 |
| Length |  |  |
| good, not burdensome | 9 | 9 |
| could have been longer | 2 | 2 |
| Fun? Yes | 7 | 7 |
| pastime | 4 | 2 |
| learn about yourself | 3 | 3 |
| make into game / competitition | 3 | 2 |
| distraction | 2 | 2 |
| simple | 3 | 1 |
| funny | 1 | 1 |
| become better | 1 | 1 |
| first few times yes | 1 | 1 |
| explanation research: good | 5 | 5 |
| Research: negative |  |  |
| schedule negative |  |  |
| (Too) busy with other things | 9 | 6 |

|  | Little variation | 4 | 2 |
| --- | --- | --- | --- |
|  | Annoying research so I rather do so | 3 | 1 |
|  | Training daily: too much | 1 | 1 |
|  | explanation research: negative / improvements |  |  |
|  | aim not clear | 7 | 4 |
|  | not good | 2 | 1 |
|  | contact information double sided n | 1 | 1 |
|  | passed by me | 1 | 1 |
|  | more explanation in practice | 1 | 1 |
|  | exhaustively formulated | 1 | 1 |
|  | Amount emails: much / too much | 6 | 5 |
|  | Reminders: no / don't remember | 2 | 2 |
|  | Reminders: not nice / obligation | 1 | 1 |
|  | Miscommunication with appointment for helping with | 1 | 1 |
|  | Length long | 3 | 3 |
|  | annoying research so I rather do something else | 3 | 1 |
|  | not a pleasant research | 1 | 1 |
|  | waste of my time | 1 | 1 |
|  | Participate more often: no |  |  |
|  | I'll need help again | 2 | 1 |
|  | Not with all the repetition | 1 | 1 |
|  | Something went wrong; daughter in law may have don | 3 | 1 |
|  | Improvements |  |  |
|  | schedule: improvements |  |  |
|  | Indicate changes in schedule more | 9 | 4 |
|  | Not on Saturday | 2 | 2 |
|  | other way communicating: group simultaneously | 4 | 2 |
|  | other way communicating: Calling (with video) more p | 3 | 3 |
|  | Reminders: improvements |  |  |
|  | Date clearer | 1 | 1 |
|  | End incorrectly set; too early | 1 | 1 |
|  | Reminders: sms | 1 | 1 |
| Evaluation computerprograms | Computerprograms (Qualtrics & Gorilla) |  |  |


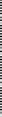

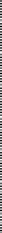

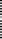

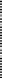

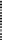

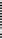

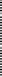

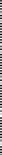

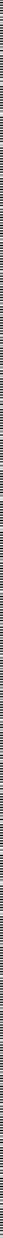

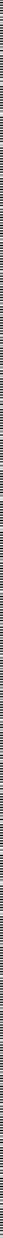


|  | Computerprograms: good 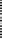 | 22 | 17 |
| --- | --- | --- | --- |
|  | progress bar good addition | 1 | 1 |
|  | Transition computerprograms |  |  |
|  | fine | 9 | 9 |
|  | went wrong in the beginning | 8 | 5 |
|  | have a good look in the beginning | 4 | 4 |
|  | Breaks |  |  |
|  | (almost) not used | 8 | 8 |
|  | annoying | 2 | 2 |
|  | not necessary | 2 | 2 |
|  | computerprograms: improvements |  |  |
|  | Instructions: you can’t go back | 3 | 2 |
|  | instructions overlap | 1 | 1 |
|  | sometimes searching for next | 1 | 1 |
| Evaluation instructions | Instructions |  |  |
|  | Instructions: positive |  |  |
|  | Instruction text: good | 21 | 12 |
|  | Instructions in a different way? No | 12 | 12 |
|  | Instructions no longer needed after 1 / 2 times | 8 | 6 |
|  | Instructions beginning a bit unclear, after fine | 8 | 5 |
|  | Instructions can be skipped but it's good they are there | 3 | 3 |
|  | Instructions: improvements |  |  |
| Adaptations instructions | you can't go back | 3 | 2 |
|  | you have to find out yourself that they stay the same | 1 | 1 |
|  | possibly different needs for elderly | 1 | 1 |
|  | part in Engels | 1 | 1 |
|  | which arrow keys? | 1 | 1 |
| Evaluation questionnaires | Questionnaires |  |  |
|  | questionnaires: negative / difficult |  |  |
|  | difficult because very variable | 2 | 2 |
|  | tiring questions | 3 | 1 |
|  | repetition questionnaires: annoying | 1 | 1 |
|  | questions confronting | 1 | 1 |


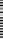

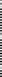

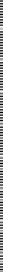

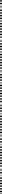

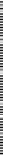

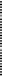

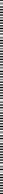

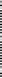

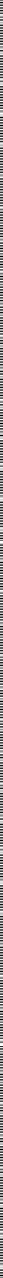

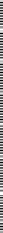

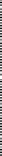

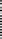


|  |  |  | pay attention |  | 1 | 1 |
| --- | --- | --- | --- | --- | --- | --- |
|  |  |  | difficult: are you doing it right? | | 1 | 1 |
|  |  | questionnaires: positive | |  |  |  |
|  |  |  | good |  | 1 | 1 |
|  |  | suggestion: ask about moments in the day | | | 1 | 1 |
| Dialysis | During dialysis | Yes | yes, a few times |  | 2 | 2 |
|  |  |  | yes, always |  | 1 | 1 |
|  |  |  | nice, already done |  | 1 | 1 |
|  |  |  | with own laptop |  | 1 | 1 |
|  |  |  |  | laptop at ward not nice | 1 | 1 |
|  |  |  | when | beginning | 1 | 1 |
|  |  | No |  | middle | 1 | 1 |
|  |  |  | keep an eye on everything | | 2 | 2 |
|  |  |  | night dialysis |  | 2 | 2 |
|  |  |  | sleep |  | 2 | 2 |
|  |  |  | small room + fast (abdominal dialysis) | | 1 | 1 |
|  |  |  | too noisy |  | 1 | 1 |
| Difference tasks | Difference measurements - training | |  |  |  |  |
|  |  | not noticed |  |  | 11 | 10 |
|  |  | noticed |  |  | 2 | 2 |
|  |  | not important |  |  | 2 | 2 |
| Evaluation possible implementa | Implementation | Positive |  |  |  |  |
|  |  |  | Implementation: good idea | | 13 | 13 |
|  |  |  | Recommend training to others: Yes | | 5 | 5 |
|  |  |  |  | Maybe it works for them | 3 | 3 |
|  |  |  |  | With evidence | 2 | 2 |
|  |  |  |  | But not for everyone (elderly) | 2 | 2 |
|  |  |  |  | Try it out with more people | 1 | 1 |

|  |  |  |  | Simple | 1 | 1 |
| --- | --- | --- | --- | --- | --- | --- |
|  |  |  | Continue training: Yes | | 4 | 4 |
|  |  |  |  | maybe it will help after all | 3 | 3 |
|  |  |  |  | with evidence | 3 | 2 |
|  |  |  |  | but in this frequency? | 2 | 2 |
|  |  |  |  | to help you | 2 | 2 |
|  |  | Adjustments |  | for evidence | 1 | 1 |
|  |  |  | Implementation: rather tablet | | 5 | 4 |
|  |  | Negative | Idea for variation |  | 1 | 1 |
|  |  |  | Implementation: not a good idea | | 1 | 1 |
|  |  |  | Recommend training to others: No | |  |  |
|  |  |  |  | I didn't improve myself | 4 | 3 |
|  |  |  |  | The research was annoying | 1 | 1 |
|  |  |  | Continue training: No | | 3 | 3 |
|  |  |  |  | I don't believe in it | 2 | 1 |
| Obstacle | ICT too difficult |  |  |  | 5 | 2 |
|  |  | Implementation: rather tablet | |  | 5 | 4 |
|  |  | On tablet / computer? | |  | 2 | 2 |
|  |  | Elderly | Computer: you have to sit down for it | | 2 | 2 |
|  |  |  |  | |  |  |
|  |  |  | Mailing system difficult for elderly | | 3 | 3 |
|  |  |  | computerprograms: possibly difficult for elderly | | 1 | 1 |
|  |  |  | Instructions: possibly different needs elderly | | 1 | 1 |
| Surroundings | Surroundings (family, friends etc.) | |  |  |  |  |
|  |  | surroundings helped? | |  |  |  |
|  |  |  | no |  | 15 | 15 |
|  |  |  | daughter / son (in law) | | 6 | 3 |
|  |  |  | spouse |  | 1 | 1 |
|  |  | surroundings opinion | |  | 16 | 15 |
|  |  | didn't share with surroundings | |  | 3 | 3 |
|  |  | surroundings not a good idea of illness | | | 1 | 1 |

| Coping / Fatigue | Fatigue symptoms |  |  |
| --- | --- | --- | --- |
|  | comorbidity | 8 | 5 |
|  | tired because of dialysis | 7 | 4 |
|  | fatigue is wearing | 2 | 1 |
|  | one has to give up so much because of fatigue | 1 | 1 |
|  | fatigue in practice | 1 | 1 |
| Idea of fatigue | Mental vs. physical fatigue | 4 | 3 |
|  | Irritation, surroundings don't realise how ill | 2 | 2 |
| Health | Health questions |  |  |
|  | Is my health not too good for this research? | 2 | 1 |
|  | Fatigue due to illness or age? | 2 | 1 |
|  | Different medication during research | 1 | 1 |
|  | Too burdensome due to ill health? | 1 | 1 |
| Motivation | Participate in research: motivation |  |  |
|  | for others | 8 | 8 |
|  | educational for you | 8 | 6 |
|  | for science | 4 | 3 |
| Health care professionals | More involvement of healthcare staff: not necessary | 22 | 18 |

Appendix C.3: Code scheme Timepoint 2 - Health care professionals

| **Codes** | **Second level code** | **Third level code** | **Fourth level code** |  | **Fifth level code** | **Quotes N** | **Pp N** |
| --- | --- | --- | --- | --- | --- | --- | --- |
| evaluation study | Positive about research | |  |  |  |  |  |
| Opinion research: positive | | | | | | | |
|  |  |  | Improvements? No (compliments) | | | 6 | 5 |
|  |  |  | hopeful / could be useful | | | 5 | 4 |
|  |  |  | charming but not burdensome | | | 1 | 1 |
|  |  |  | important because fatigue is common in patients | | | 1 | 1 |
|  |  | Participate again: yes | | |  | 1 | 1 |
|  |  |  | contribute to science |  |  | 2 | 2 |
|  |  |  | little research is done | | | 2 | 1 |
|  |  |  | valuable for patients |  |  | 2 | 2 |
|  |  |  | not burdensome |  |  | 1 | 1 |
|  |  |  | depends on results this research | | | 1 | 1 |
| recruitment |  |  |  |  |  |  |  |
| recruitment: positive | | | | | | | |
|  |  |  | recruitment not burdensome | | | 5 | 4 |
|  |  |  |  |  | dialysis patients already come to the hospital | 1 | 1 |
|  |  |  | recruitment in this way good | | | 4 | 3 |
|  |  |  | connection with patients | | | 1 | 1 |
| obstacles |  | obstacles / improvements | | |  |  |  |
| recruitment |  | obstacle | ICT limits recruitment | | | 7 | 4 |
|  |  | obstacle | misconception about fatigue symptoms patient - caretaker | | | 5 | 3 |
|  |  | obstacle | multiple studies simultaneously; burdensome for patients | | | 2 | 2 |
|  |  | improvement | don't take just one startmoment | | | 2 | 1 |
|  |  | improvement | involve peritoneal earlier | | | 2 | 1 |
|  |  | obstacle | you have to think about it during work | | | 2 | 1 |
|  |  | obstacle | burdensome in combination with regular work | | | 1 | 1 |
|  |  | obstacle | there has to be room for it in the conversation | | | 1 | 1 |
|  |  | improvement | improvement for self: let the researcher call the patient | | | 1 | 1 |
|  |  | improvement | miscommunication about locations | | | 1 | 1 |
|  |  | obstacle | patients too busy with other things | | | 1 | 1 |

| role current study |  | recruitment |  |  | 4 | 3 |
| --- | --- | --- | --- | --- | --- | --- |
|  |  | recruitment + contact researcher | |  | 3 | 3 |
| suggestions | implementation | good idea |  |  |  |  |
| for |  |  |  |  | 6 | 6 |
| implementation |  | at nephrology department | |  |  |  |
|  |  |  | feedback with caretaker | | 5 | 2 |
|  |  |  | help from nurse during dialysis | | 3 | 3 |
|  |  |  | social worker |  | 3 | 1 |
|  |  |  | instructions during outpatient visit | | 3 | 1 |
|  |  |  | involve family |  | 1 | 1 |
|  |  |  | during dialysis or at home | | 1 | 1 |
|  |  |  | during dialysis is possible | | 1 | 1 |
|  |  | patient group |  |  |  |  |
|  |  |  | widely applicable |  | 8 | 4 |
|  |  |  | nott in first phase dialysis | | 1 | 1 |
|  |  |  | not too old, not too terminal | | 1 | 1 |
|  |  | recommendations |  |  |  |  |
|  |  |  | app better accessible | | 5 | 3 |
|  |  |  | helpdesk |  | 5 | 2 |
|  |  |  | clear instructions for hospital personel | | 3 | 2 |
|  |  |  | make instruction video | | 2 | 2 |
|  |  |  | online / website |  | 2 | 2 |
|  |  |  | information folder |  | 1 | 1 |
|  |  |  | clear instructions |  | 1 | 1 |
|  |  | recruitment |  |  |  |  |
|  |  |  | fellow sufferers meeting | | 3 | 1 |
|  |  |  | presentation during theme-night | | 2 | 1 |
|  |  |  | recruitment via patient association | | 2 | 2 |
|  |  |  | caretaker can name it, other than that up to patient | | 1 | 1 |
|  |  | own role |  |  |  |  |
|  |  |  | introduce |  | 3 | 2 |
|  |  |  | don't know yet |  | 1 | 1 |

| obstacles |  |  |
| --- | --- | --- |
| ICT difficult for some people | 12 | 5 |
| know of existence hospital personel | 5 | 3 |
| other cultures | 2 | 2 |
| too little time at outpatient care | 2 | 1 |
| willingness nurses | 1 | 1 |
| money | 1 | 1 |
| low health skills | 1 | 1 |
| researcher from the outside | 1 | 1 |
| solutions |  |  |
| key users @ nurses | 4 | 3 |
| substantiate with results | 1 | 1 |
| put reminder in caretakers system | 1 | 1 |
| contact with patient about study? |  |  |
| no / little | 5 | 5 |
| useful? Not burdensome | 2 | 1 |
| dropouts | 1 | 1 |
| patient thought it was a lot | 1 | 1 |
| hopeful in advance | 1 | 1 |
| contact with colleagues about study? |  |  |
| Yes |  |  |
| Yes, agreeing | 2 | 2 |
| recruitment / if patient named it | 2 | 2 |
| No | 2 | 2 |
